# Effects of bird-feeding activities on the health of wild birds

**DOI:** 10.1093/conphys/cov058

**Published:** 2015-12-21

**Authors:** Travis E Wilcoxen, David J Horn, Brianna M Hogan, Cody N Hubble, Sarah J Huber, Joseph Flamm, Madeline Knott, Lisa Lundstrom, Faaria Salik, Samantha J Wassenhove, Elizabeth R Wrobel

**Affiliations:** af1 Biology Department, Millikin University, 1184 West Main Street, Decatur, IL 62522, USA; af2 Natural Resources Conservation Service, United States Department of Agriculture, 2623 Sunrise Drive, Springfield, IL 62703, USA; af3 Department of Poultry Science, University of Georgia, 215 Poultry Science Building, Athens, GA 30602, USA

**Keywords:** Anthropogenic food, avian physiology, disease, songbirds

## Abstract

Among the most popular reasons that people feed wild birds is that they want to help birds. The extent to which supplemental food helps birds, however, is not well established. From spring 2011 to spring 2014, we examined how feeding of wild birds influences the health of individual birds at forested sites in central Illinois, USA. Specifically, we compared three forested sites where we provided supplemental food with three forested sites for which no supplemental food was available and monitored changes in the individual health of birds. In addition, we determined whether any changes in bird health had occurred after feeders had been removed from sites 10 months before. Generally, the individual health of birds improved with supplemental feeding, including increased antioxidant levels, reduced stress (heterophil-to-lymphocyte ratio) and more rapid feather growth. In some species, we also found improved body condition index scores and innate immune defense. The difference among sites was not present 10 months after feeders were removed, suggesting that the impact on health was indeed related to supplemental feeding. Potential negative effects of supplemental feeding were also found, including an increase in infectious disease prevalence among individual birds at forested sites where supplemental food was offered. Birds with clear signs of pathology showed deficits in most of the physiological metrics in which birds at feeder sites typically showed improved health condition. At the peak of prevalence of infectious disease, 8.3% of all birds at feeders exhibited symptoms of conjunctivitis, pox, dermal disease or cloacal disease. We found both positive and negative impacts of wild bird feeding, and that, in general, birds that had access to supplemental food were in better physiological condition. Moreover, the negative effects we found may be mitigated by hobbyists engaging in safer bird-feeding practices.

## Teaser

We completed a three-year study of 11 bird species investigating the effects of bird feeding on the health of wild birds. Birds at sites with feeders were generally in better overall health than birds at sites without feeders, although birds at sites with feeders had a greater prevalence of disease.

## Introduction

The provisioning of anthropogenic food to wild birds is a popular, yet understudied wildlife conservation issue with limited regulations in the USA. In 2011, 52.8 million Americans over the age of 16 years fed birds and other wildlife around their homes and spent over $5 billion on bird food, feeders, houses, baths and other accessories (US Fish and Wildlife Service, 2012). Previous studies of impacts of bird feeding on wild birds have largely focused on individual species, despite the consistent interaction of multiple species within a community at feeders. Many published studies of bird feeding to date have focused on seed and feeder preferences (Geis, 1980; Horn *et al*., 2014; Johansen *et al*., 2014), disease transmission (Dhondt *et al*., 1998), population trends, range expansions, irruptive migrations of birds at feeders (Hochachka *et al*., 1999; Bonter and Harvey, 2008; Robb *et al*., 2008) and the effects of feeders on urban bird community structure (Galbraith *et al*., 2015). One of the most comprehensive reviews of the impacts of bird feeding on bird populations was published by Robb *et al*. (2008), in which a meta-analysis revealed that supplemental feeding across studies of diverse avian taxa has led to either improved or unaffected breeding success in nearly all cases, with only a few exceptions of reported negative impacts of supplemental feeding on breeding.

Three of the more pressing issues in bird-feeding research are the link between bird-feeding activities and transmission of diseases among birds (e.g. Bradley and Altizer, 2006), the degree to which anthropogenic food serves as a dietary supplement to a diverse array of food items consumed by birds and the degree to which feeders create dependency among bird populations (Brittingham and Temple, 1992; Jones and Reynolds, 2008). Each of these issues, disease transmission in particular, has been considered in existing studies of the impacts of anthropogenic food on wildlife. A recent meta-analysis by Becker *et al*. (2015) includes excellent evidence-based discussion of the importance of fully evaluating the costs and potential negative impacts of human alteration of the foraging ecology of wildlife and its link with increased disease transmission. Given that some studies show positive impacts of bird-feeding activities, whereas other studies show costs or negative impacts, combined with the knowledge that most bird populations are in decline, it is absolutely crucial that all investigations of the impacts of anthropogenic food on bird health consider benefits and costs alike.

Habitat alteration and destruction undoubtedly impose the greatest human impacts on bird populations, and many species of birds are in decline worldwide (Butchart *et al*., 2010). The large amounts of anthropogenic food provided to wild birds may be as influential as the habitat changes associated with human development and land-management practices (Amrhein, 2013). Some conservation organizations, such as the Cornell Laboratory of Ornithology (USA) and the British Trust for Ornithology (UK), actively promote human provisioning of food to wild birds, whereas others, such as BirdLife Australia, are far more cautious with their recommendations for human–wildlife interactions (Jones, 2011). Regardless of the formal stance of avian conservation organizations, these recommendations are not based on empirical work, because sufficient evidence of the effects of anthropogenic food on the physical health of wild birds is lacking.

Several fundamental questions about wild bird feeding remain. In particular, few studies have examined the impact of supplemental food on wild bird populations, including how bird feeding influences the health and energy demands of individual birds and may change the overall bird community (although see Brittingham and Temple, 1988; Geis and Pomeroy, 1993; Pravosudov *et al*., 2001; Schoech *et al*., 2004). From spring 2011 to spring 2014, we examined how bird feeding impacts wild birds by evaluating the health of individual birds with a broad range of metrics, including body condition, stress, antioxidant levels, nutritional condition, immune function and disease, by comparing forested sites with and without feeders.

Defining avian health and choosing relevant metrics can be challenging. In many cases, physiological responses are context dependent, and in general, it is unlikely that any single measure is truly representative of the health of a free-living bird. For this reason, we used multiple metrics that measure a diverse array of physiological functions. Body condition has been defined in a very broad sense to indicate the physical make-up of a bird that confers the ability of an individual to cope with present and future physiological stress, and therefore, the ability to enhance fitness (Carrascal *et al*., 1998). Mass alone is unlikely to serve as a reliable indicator of condition, as an animal can be heavy because it is structurally large or because it is carrying abundant fat or protein (Dobson, 1992); therefore, using measures that consider the structural size and mass, as well as storage of macronutrients such as fat, are important. In general, wild songbirds have very little fat because they need to remain light. However, fat reserves can be important in buffering an animal against fluctuations in food supply or serving as fuel for energetically demanding flight, as in migrating birds (Blem, 1990).

Haematology is presumed to provide useful indicators for assessment of the health and nutritional condition of animals (Averbeck, 1992). Many biomarkers can be obtained from blood samples and have served as reliable indicators of physiological condition in multiple studies of avian physiology. Reproductive hormones are good indicators of reproductive condition. In males, testosterone concentrations below the normal range could lead to an inability to maintain sexual characteristics, attract mates, defend territories and reproduce (Wingfield, 2003). Testosterone concentrations beyond the high end of the normal range may enhance each of those features over the short term, but they can also reduce longevity (Schroderus *et al*., 2010). Likewise, estradiol levels outside of the normal range pose similar problems for female birds (Wingfield and Farner, 1978). Other markers may serve as more direct indicators of a bird's overall health. Total plasma protein concentrations below the normal range can indicate poor nutritional condition and concentrations above the normal range can appear following stress responses or dehydration (Allison, 1955). Circulating antioxidant concentrations are excellent indicators of the ability to resist oxidative stress and oxidative damage associated with normal metabolic processes and stressful stimuli that instigate physical exertion (Cohen *et al*., 2009). Leucocyte differentials can serve as indicators of recent immune challenges as well as immune preparedness, but elevated ratios of heterophils to lymphocytes, for instance, can serve as an indicator of chronic stress. Finally, blood samples can be used in functional immunological assays to estimate an organism's ability to resist microbial infection, with poor responders being considered susceptible to infection and strong responders serving as indicators of high-quality individuals capable of maintaining costly defenses (Millet *et al*., 2007).

Each bird completes at least one moult per year, dropping each of its feathers and growing a new one in its place. During the regrowth process, a visible growth bar is developed with each day of growth until the feather is fully developed. The assessment of feather growth bar length, or ptilochronology, has been validated in captive and wild birds as a reliable indicator of nutritional condition (Grubb, 1989). Therefore, is likely to serve as a good measure of the impacts of anthropogenic food on bird nutritional condition.

The main objective of the present study was to use a broad range of metrics to assess the impacts of bird feeding on the health of wild birds. We hypothesized that supplemental feeding would lead to differences in the health of birds at sites with supplemental food available compared with birds at sites without supplemental food. Specifically, we predicted that birds at sites with constant, predictable birdseed at feeders would be in better overall health than birds at sites without feeders. In addition, we predicted that the multiyear, multispecies approach of our study would reveal important physiological costs to the use of anthropogenic food by wild birds.

## Methods

### Study areas

Six forested sites in central Illinois, USA were used, with each site having limited, if any, bird-feeding activity before the study. The sites were Robert Allerton Park (University of Illinois, Piatt County), Fort Daniel Conservation Area [Macon County Conservation District (MCCD), Macon County), Friends Creek Conservation Area (MCCD, Macon County), Rock Springs Conservation Area (MCCD, Macon County), Sand Creek Conservation Area (MCCD, Macon County) and Valentine Park (Piatt County Forest Preserve, Piatt County) with adjacent private property (Piatt County). Each of the six study sites was placed into one of three categories based on the size and composition of forest. Fort Daniel and Valentine Park with adjacent private property were grouped together because they are the smallest sites at 80 and ∼50 ha, respectively. Friends Creek and Rock Springs are the largest sites and have older forests and open spaces, with a mixture of young forest dominated by maples and honeysuckle and mature forest with oak, hickory and walnut and have 211 ha and 526 ha, respectively. Allerton and Sand Creek are larger sites that have a greater proportion of mature hardwood forest and open spaces with 607 ha and 303 ha, respectively.

### Feeder set-ups

During spring 2011, no feeders were present at any sites and birds were captured, banded, measured and released to record baseline physiological metrics before manipulation of food availability. After collecting baseline metrics at all sites, bird feeders were added to three of the six sites (Fort Daniel, Rock Springs and Sand Creek) in June 2011, while the remaining three sites were used as control sites with no supplemental food provided. Study sites were paired based on site characteristics described in the section above and were randomly assigned as either feeder or non-feeder sites within those pairs. During 2011, birdseed was provided by The Scotts Company, whereas during 2012 and 2013, birdseed was provided by Siemer Enterprises. The composition of the seed blend used in 2012 and 2013 was 50% black-oil sunflower, 18% white proso millet, 10% safflower, 9% whole peanuts, 7% medium sunflower chips and 6% red proso millet. A similar blend was used in 2011. In general, feeders were filled two or three times per week as needed over the course of the study so that birdseed was always available.

Four bird feeders were set up as part of a single bird-feeding station at the three sites with bird feeders. The feeders included a ‘Going Green’ extra-large premier hopper feeder, a ‘Going Green’ fly-through platform feeder and two brushed copper six-port tube feeders. Each feeder was individually mounted by pole, with each pole containing a raccoon baffle. All feeders and hardware were manufactured by WoodLink Ltd (Mount Ayr, IA, USA). The feeders were set up 2 m apart from one another in a straight line. Feeding stations were established in both grassy areas adjacent to forests (e.g. Sand Creek) or within the forests themselves (e.g. Fort Daniel and Rock Springs). Feeders were removed in June 2013, leaving ∼10 months between the last date when supplemental food was provided and the start of our final sampling period in April 2014.

### Study species

From 8 April 2011 to 2 June 2014, we captured 1680 birds from the six study sites, which included 542 (32.3%) birds that were recaptured during our sampling period. Of those recaptured, 15.2% of the recaptures occurred in 2011, 25.9% of the recaptures were from 2012, 44.4% of the recaptures were from 2013, and 14.5% of recaptures were from 2014 (10 months after feeders were removed). Eleven species that commonly use feeders were captured at a frequency great enough across multiple sites, each with feeders and without feeders, to warrant inclusion in statistical analysis, representing 1510 captures that were included in the analysis, as follows: American goldfinch (*Carduelis tristis*; *n* = 124), black-capped chickadee (*Poecile atricapillus*; *n* = 208), brown-headed cowbird (*Molothrus ater*; *n* = 150), chipping sparrow (*Spizella passerina*; *n* = 86), downy woodpecker (*Picoides pubescens*; *n* = 102), gray catbird (*Dumetella carolinensis*; *n* = 102), house finch (*Haemorhous mexicanus*; *n* = 101), indigo bunting (*Passerina cyanea*; *n* = 124), northern cardinal (*Cardinalis cardinalis*; *n* = 193), tufted titmouse (*Baeolophus bicolor*; *n* = 180) and white-breasted nuthatch (*Sitta carolinensis*; *n* = 140). In many analyses, data were available from all 11 species. However, that was not the case for all analyses because of limitations in blood sample size associated with variation in the size of birds, funding available to analyse all samples, and in cases such as feather growth bar measurement, the ability to see growth bars on feathers. All 11 species were used in the following analyses unless otherwise stated.

### Bird health assessment

#### Capture and sampling

We captured birds in mist nets at each of the six sites. Each captured bird was given a uniquely numbered United States Fish and Wildlife Service aluminum leg band. The capturing of birds using mist nets took place from spring 2011 to spring 2014 with the exception of winter 2011, autumn 2012 and winter 2012. We did not capture birds during the winter because winter temperatures are frequently below 0°C, and we did not capture bird on any days when air temperature was below 5°C to avoid the detrimental effects of cold stress on bird physiology. We did not capture birds in autumn 2012 because of funding limitations. During each sampling day, six to ten mist nets were set up in the same general locations, and all birds were captured between 05.15 and 11.15 h.

We assessed the following seven aspects of bird health: stress, body condition, antioxidant concentrations, feather quality (as an indicator of nutritional condition), reproductive physiology, immune function and disease. For those measures of health that required blood, blood samples were collected from individuals upon capture. Blood was collected in microhaematocrit capillary tubes following venipuncture of the brachial vein with a 25 or 27½ gauge needle (Romero and Romero, 2002; Schoech *et al*., 2007). The blood samples were stored on ice in coolers in the field until return to the labortory, where the tubes were spun in a microhaematocrit centrifuge to separate whole blood from plasma. The plasma was drawn off with a 100 µl Hamilton syringe, and stored frozen in plastic vials at −20°C until future use in assays. A second blood sample was sometimes collected for the *in vitro* microbial killing assay after ensuring sterility by liberally swabbing the area around the brachial vein with 70% alcohol and allowing it to air dry for 10–15 s. We used a 100 µl pipette and sterile tip to transfer 30 µl of whole blood from a sterile capillary tube to a screw-cap Eppendorf vial that contained 300 µl of CO_2_-independent media with 4 mM l-glutamine. The total volume of blood collected was below the recommended limits of <1% of total blood volume (McGuill and Rowan, 1989).

#### Heterophil-to-lymphocyte ratio

We used white blood cell counts as a measure of stress in feeder-using species. Specifically, we counted the ratio of heterophils to lymphocytes (Gross and Siegel, 1983; Campo and Davila, 2002). Heterophils require less energy to produce, whereas lymphocytes require more energy to produce and maintain. Therefore, birds that are stressed and are allocating energy to surviving stressful conditions produce more heterophils relative to lymphocytes. To measure the heterophil-to-lymphocyte (H:L) ratio, blood smears were made in the field by placing a single drop of blood onto a microscope slide, smearing the droplet, allowing it to dry, and fixing it to the slide with methanol. The slide was later stained with Wright–Giemsa stain and examined under a compound microscope at a magnification of ×400 with oil immersion. We identified cells using published avian guidelines (Dein, 1986; Campbell, 1988). Heterophils and lymphocytes were counted on the slide in multiple fields of view until the combined count of both cell types reached 100 cells.

#### Fat

We also used an assessment of fat stores in the birds by recording a score (increments of one from −1 to 2, with −1 being no fat and protruding keel bone to 2 being globular fat deposits) of the amount of subcutaneous fat stored at the furculum. This scale is consistent with the methods of Mueller and Berger (1966). T.E.W. was present for all captures and assessments of fat score.

#### Antioxidants

We used an OxiSelect™ Total Antioxidant Capacity (TAC) assay kit, which was purchased from CellBioLabs, Inc. (San Diego, CA, USA), to determine the total antioxidant capacity. This assay is based upon the reduction of copper (II) to copper (I) by biological samples. In this study, the assay was completed with 20 µl of plasma from each bird as well as known concentrations of uric acid standards, both of which were pipetted into separate wells on a 96-well microplate. Both the samples and the standards were diluted with a reaction buffer. Then a copper ion reagent was added to the reaction, followed by a 5 min incubation period. Using a stop solution prepared in laboratory, the reaction was halted after the 5 min had passed. The colorimetric test was completed by reading the wells in the plate at 492 nm using a spectrophotometric microplate reader (BioRad iMark; BioRad Laboratories, Inc., Hercules, CA, USA). The total antioxidant capacity of the plasma samples (copper-reducing equivalents) was determined by making comparisons to a uric acid standard curve generated with known concentrations. The normal range of uric acid and copper-reducing equivalent values for birds is from 0.22 (750 µM copper-reducing equivalents) to 0.93 mM (2035 750 µM copper reducing equivalents; Tsahar *et al*., 2006).

#### Haematocrit

We determined the haematocrit for each bird, which is the ratio of red blood cells to plasma following centrifugation of the blood samples in microhaematocrit capillary tubes. The haematocrit is considered the most reliable measure of red blood cell numbers and is both a good indicator of the ability to nourish the body with oxygen and a measure of hydration (Dein, 1986). Values between 35 and 55% are considered normal for birds (Campbell, 1988). Low values of haematocrit are also indicative of bacterial infections and gastrointestinal disorders, including parasitism and haemorrhage (Dein, 1986), or may reflect nutritional deficiencies of minerals, such as iron or copper (Campbell, 1988).

#### Total plasma protein

We estimated the total amount of protein in each bird's blood by determining the optical density of the liquid portion of blood (plasma). We used a hand-held refractometer with ∼10 µl of plasma from each captured individual to determine total protein content of the plasma. Circulating concentrations of protein in the blood are thought to be an index of total protein reserves in an animal (Allison, 1955), and the normal range for birds is 2.5–4.0 mg/dl (Fair *et al*., 2003). In general, plasma protein composition involves albumin and clotting proteins, but many of the plasma proteins are also immunoglobulins, and other studies have found that refractometry is a good predictor of serum immunoglobulin concentration (Fair *et al*., 2003).

#### Reproductive hormones

We used plasma samples from the spring and summer seasons, when birds were either coming into, or in, reproductive condition, for the assessment of reproductive hormone concentrations. We first validated enzyme immunoassay (EIA) kits from Enzo Life Sciences (Farmingdale, NY, USA) for testosterone (ADI-900-065) in samples from male birds and for estradiol (17β-estradiol high sensitivity; ADI-900-174) in samples from female birds, for each of the 11 species. We used pooled plasma samples in 10, 20, 30 and 40 µl volumes, using appropriate amounts of assay buffer in each case to ensure that the total volume in each well was the same for each sample and running each in duplicate to optimize the assays for each species. Beyond the sample volume adjustments, the remainder of each assay was completed following the manufacturer's recommendations. Final hormone concentrations for the samples were determined by comparing the values from the average of duplicate samples with a nine-point standard curve generated by the manufacturer's provided standards. Intra-assay coefficients of variation (CVs) were calculated from six standard samples included in each plate, yielding average CVs of 7.21% among the plates and intra-assay CVs of 11.35% for testosterone and 8.89% for estradiol.

#### Body condition

We used a traditional body condition index with a ratio of structural size to mass to assess overall body condition (Green, 2001). The index was generated for each bird using a principal components analysis of structural measures (lengths of the left wing cord, tail and left tarsus), extracting principal component one (PC1; average variance explained = 84.6%), regressing mass against PC1, and using the residual values as the body condition index (Green, 2001; Wilcoxen *et al*., 2010).

#### Innate immune defense

We used three microbial killing assays to test the effects of bird feeding on the ability of birds to resist infection (innate immune function) to the following three different infectious microbes: *Escherichia coli*, a potential Gram-negative intestinal pathogen; *Staphylococcus aureus*, a Gram-positive bacteria and the causative agent of skin infections and respiratory infections; and *Candida albicans*, a common yeast-like fungal pathogen that can cause infection of the mouth, respiratory tract or cloaca. We followed the procedures outlined by French and Neuman-Lee (2012) for whole blood, with minor modifications described below, and performed the assays in a sterile working environment. We completed this *in vitro* challenge by inoculating 110 µl of the whole blood and cell media mix (prepared in the field) with one of the three pathogens (10 µl of solution at ∼250 colony forming units) and completed the challenge of humoral immunity at 41°C (avian body temperature) for 20 min with the *E. coli* challenge and 40 min each for *S. aureus* and *C. albicans* challenges. We added 50 µl of the mixture to 100 µl of sterilized tryptic soy agar in duplicate wells on a 96-well plate, and placed each sample for incubation at 37°C, the ideal growth temperature for each microbe. Control wells were also included to represent maximal growth without immune inhibition, and these control samples included only the cell media and the pathogen inoculate, but were otherwise subjected to the same protocol. To calculate the microbial killing ability for each sample, we first subtracted the initial, background absorbance readings taken before incubation from 24 h absorbance readings for *E. coli* and *S. aureus* samples and 48 h absorbance readings for *C. albicans.* The percentage of microbes killed was calculated as one minus the mean absorbance for each sample (samples were run in duplicate), divided by the mean absorbance for the controls (also in duplicate), then multiplied by 100 (French and Neuman-Lee, 2012).

From the collection of values of microbial killing ability, we generated a microbial killing ability index by using a principal component analysis to reduce the values into two principal components. When combined, these principal components accounted for 74.8% of the variance, and therefore, the combined value was used as the microbial killing ability index.

#### Nutritional condition

With each capture (excluding woodpeckers), we collected the outermost right rectrix and measured the following: (i) growth bars from feathers in each spring for feathers that were grown in each of the previous autumn seasons; and (ii) for feathers from birds that were captured and later recaptured, where an original feather was collected, its growth bars were compared with the induced feather that grew back and was collected upon subsequent capture. For both the original and induced feathers, we measured the breadth (in millimetres) of 10 adjacent daily growth bars and divided the sum by 10 to determine average daily growth (for method, see Grubb, 1989). To standardize our measure of feather growth in order to control for the size of the sampled birds, we divided the average daily growth of the induced feather by the average daily growth of the original (Grubb, 1989).

#### Disease

We examined the birds for the prevalence of four different pathological conditions and compared the prevalence between feeder and non-feeder sites. The four diseases for which we looked for outward expression of the disease were as follows: conjunctivitis (i.e. pink eye); avian pox; fungal skin disease; and cloacal infections. Although we are uncertain of the incidence of asymptomatic birds in the population, we did capture birds with clear signs of infection. Birds were considered to be inflicted with conjunctivitis when their eyes or inner throat were pink and swollen. Birds were also examined for pox lesions around the beak, legs and underwing. Fungal skin diseases were detected by missing downy feathers inconsistent with the timings or patterns of moult and often accompanied by skin discoloration. Finally, birds with bright green discharge and cloacal swelling inconsistent with normal cloacal morphology were considered to be inflicted with a pathogen.

### Statistical analysis

All statistical analyses were completed using SPSS 22.0 (2014; IBM, Inc.) To assess the health impacts of bird feeding, we used nine separate general linear mixed models (LMMs) with each of the health metrics as a dependent variable in each model and species (each of the 11 species listed above), sex (male, female, juvenile/unknown or adult/unknown), treatment (feeders or no feeders), bird age (hatch year, second year, after hatch year or after second year), year (2011, 2012 or 2013) and disease status (1 = showing symptoms of disease or 0 = without symptoms of disease), as well as two- and three-way interactions as independent variables. We included the site identity and bird identity (USGS band number) as random variables to control for potential non-independence in our data that might result from sampling repeatedly from the same sites and from unbalanced recapturing of birds. The random variables explained a significant amount of variance in all mixed model analyses (Wald *Z* > 12.401, *P* < 0.001 in all cases) and were retained in all models. Owing to the use of multiple comparisons, we used a Bonferonni correction, reducing our α (*P*-value for acceptance of statistical significance) to 0.006 for all initial models and set an α of 0.001 for all follow-up models completed to address significant interactions. All means and standard errors reported below and represented in the figures are estimated marginal means (EMMs) and standard errors (SEs) derived from the general linear mixed model analysis.

We also had a number of birds from which we had collected the outermost right retrix that were recaptured before their next moult, which gave us the opportunity to compare the growth bar lengths on the initial feather with the growth bar lengths on the feather that was induced by our removal of the original feather. Feather samples from all seasons were included in the analysis to ensure inclusion of all recaptures prior to their next moult. For this analysis, we used a repeated measures ANOVA (RMANOVA) with growth bar length as the dependent variable, capture time (initial and recapture) as the within-subject variable and species, treatment, sex and age as between-subject variables.

We used a generalized linear mixed effects model (GLM) with a binomial distribution on the number of birds showing signs of disease vs. those without symptoms to determine whether access to feeders significantly influenced the likelihood of birds contracting a disease. The model was otherwise structured as the general linear mixed model described above, with species, sex, treatment, bird age, year and two- and three-way interactions as fixed factors. Again, site and bird identity were included as random variables.

## Results

### Heterophil-to-lymphocyte ratio

**Figure 1:**
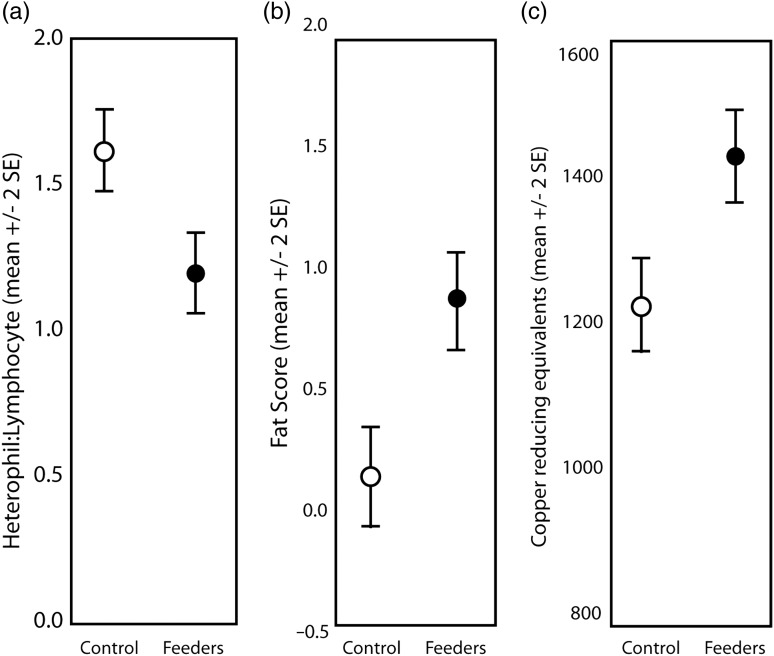
Physiological differences in 11 bird species at sites with feeders and sites without feeders, sampled in Central Illinois from 2011 to 2014. Shown here are heterophil-to-lymphocyte ratio means (**a**), fat score means (**b**) and total antioxidant capacity (copper-reducing equivalent) means (**c**). The differences were not year specific or species specific; therefore, all years and species are combined in this figure. Differences are statistically significant in all comparisons shown here.

Although there was significant variation in H:L among species (*F*_10,1109_ = 16.551, *P* < 0.001; online supplementary material TableS1), the effects of feeders on H:L were species independent (non-significant species × treatment interaction, *P* = 0.810). There was a significant main effect of treatment on H:L ratios, with birds at sites with feeders having significantly lower H:L ratios (EMM = 1.27 ± 0.13 SE) than birds at sites without feeders (EMM = 1.67 ± 0.14 SE; *F*_1,1109_ = 18.204, *P* < 0.001; Fig. 1a). There was also a significant main effect of disease on H:L ratios (see ‘*Disease*’ section below). There were no other significant interactions or main effects with regard to H:L ratios (*P* > 0.007 in all cases).

### Fat

There was a significant main effect of treatment on fat score (*F*_1,1279_ = 15.151, *P* < 0.001; Fig. 1b) and significant variation in fat score among species (*F*_10,1279_ = 5.650, *P* < 0.001), but the effects of treatment on fat score was not species specific (non-significant species × treatment interaction, *F*_10,1186_ = 0.697, *P* = 0.728). Birds at sites with feeders had significantly greater fat scores (EMM = 0.89 ± 0.15 SE) than birds at sites without feeders (EMM = 0.25 ± 0.15 SE). There were no other significant interactions or main effects with regard to fat score (*P* > 0.057 in all cases; online supplementary material Table S2).

### Antioxidants

We found a significant main effect of treatment on total antioxidant capacity (*F*_1,1221_ = 35.843, *P* < 0.001) independent of species (non-significant treatment × species interaction, *F*_10,1096_ = 0.918, *P* = 0.515). Birds at sites with feeders had significantly greater total antioxidant capacity (EMM = 1477.28 ± 23.24) than birds at sites without feeders (EMM = 1240.32 ± 24.98; Fig. 1c). There was a main effect of species (*F*_10,1221_ = 5.449, *P* = 0.001) and a main effect of disease (see ‘*Disease*’ section below). There were no other significant interactions of main effects (*P* > 0.051 in all cases; online supplementary material Table S3).

### Body condition

**Figure 2:**
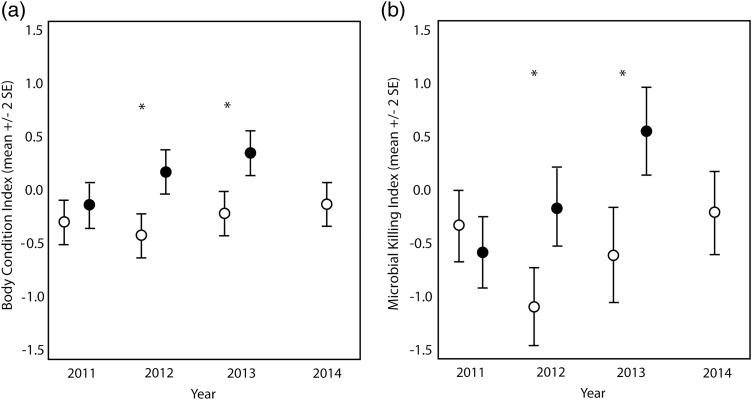
Body condition index means (**a**) and microbial killing ability index means (**b**) from 11 bird species at sites with feeders and sites without feeders in central Illinois from 2011 to 2014. *Significant difference (*P* < 0.001).

There was a significant two-way interaction of treatment by year for body condition index (*F*_2,1382_ = 6.356, *P* = 0.001), which shows that the effect of feeding on body condition was dependent upon year. Following the significant interaction, we ran separate LMMs within each year. We found no significant difference in body condition between birds at sites with feeders and birds at sites without feeders in 2011 (*P* = 0.534). Birds at sites with feeders were in significantly higher body condition than birds at sites without feeders in 2012 (*P* < 0.001) and in 2013 (*P* < 0.001; Fig. 2a). There was also a significant main effect of species (*F*_10,1382_ = 3.748, *P* = 0.002) and a significant main effect of disease (see ‘*Disease*’ section below). There were no other significant interactions or main effects (*P* > 0.042 in all cases; online supplementary material Table S4).

### Innate immune defense

For the microbial killing assay index, there was a significant two-way interaction of treatment and year (*F*_2,1020_ = 5.488, *P* = 0.005). Owing to this significant interaction, we ran separate LMMs within each year and found that birds at sites with feeders had significantly greater microbial killing ability in 2012 (*P* < 0.001) and in 2013 (*P* < 0.001) than birds at sites without feeders, but there was no difference between sites with feeders and sites without feeders in 2011 (*P* = 0.209; Fig. 2b). We also found a significant main effect of disease (see ‘*Disease*’ section below). There were no other statistically significant interactions or main effects not involved in an interaction (*P* > 0.038 in all cases; online supplementary material Table S5).

### Haematocrit

The only variable to have a significant relationship with haematocrit was species (*F*_10,900_ = 2.590, *P* < 0.001), which simply shows that there is significant variation in haematocrit levels among species. Disease did not have a significant effect on haematocrit (*F*_1,900_ = 2.632, *P* = 0.105). All other main effects and interactions were not significant (*P* > 0.125 in all cases; online supplementary material Table S6).

### Total plasma protein

The only variable to have a significant relationship with total plasma protein was disease (see ‘*Disease*’ section below). No other interactions or main effects were statistically significant (*P* > 0.057 in all cases; online supplementary material Table S7), suggesting that total plasma protein was not influenced by bird feeding.

### Reproductive hormones

For the reproductive hormone analysis, we had sufficient volumes of plasma to complete the assays only for brown-headed cowbird, downy woodpecker, gray catbird and northern cardinal. We found no significant three- or two-way interactions or main effects for male birds with regard to testosterone concentrations (*P* > 0.830 in all cases; online supplementary material Table S8).

We found no significant interactions or main effects with regard to female estradiol concentrations (*P* > 0.095 in all cases; online supplementary material Table S9). In general, bird feeding does not appear to influence reproductive hormone concentrations in this context.

### Nutritional condition

**Figure 3:**
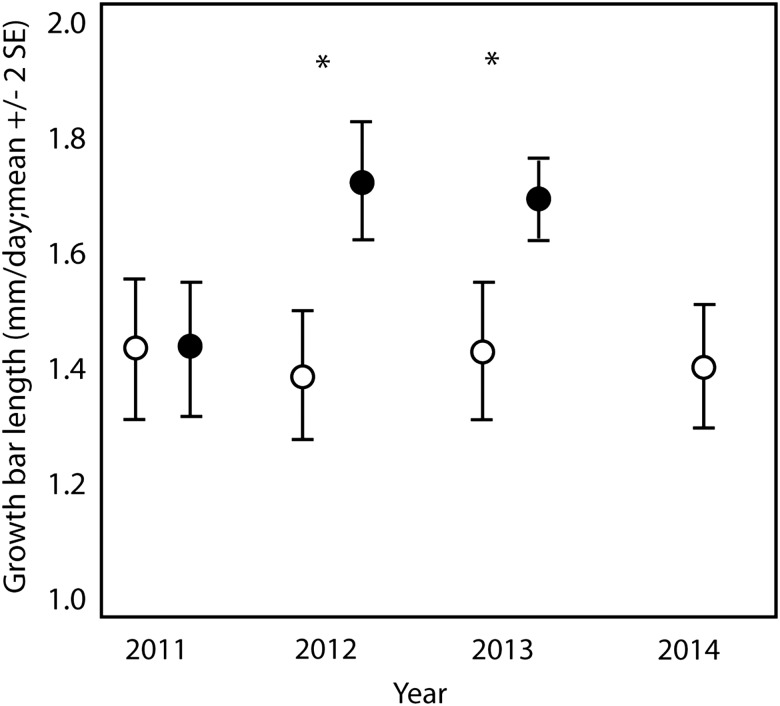
Difference in mean growth bar length for six species at sites with feeders and sites without feeders in central Illinois from 2011, when feathers were grown before feeders were available at any sites, from 2012 and 2013, when feathers were grown with some birds having access to feeders and other birds at sites without feeders, and from 2014, when feathers were grown at sites with feeders removed 10 months before and sites with no bird-feeding history. The differences were not species specific; therefore, data from all six species are combined in this figure. *Significant difference (*P* < 0.001).

We examined feather growth bar length in the following six species, for which viewing and measuring growth bars was possible: black-capped chickadee, gray catbird, house finch, indigo bunting, northern cardinal and tufted titmouse. We found a significant interaction between treatment and year (*F*_4,985_ = 3.882, *P* = 0.004). Separate LMMs within each of the years revealed no difference in growth bar length between birds at sites with feeders and birds at sites without feeders in 2011 (*P* = 0.695). Birds at sites with feeders had greater growth bar lengths than birds at sites without feeders in 2012 (*P* = 0.001) and in 2013 (*P* < 0.001; Fig. 3). There was also a significant main effect of species (*F*_6,998_ = 106.423, *P* < 0.001). There were no other significant interactions or main effects not involved in an interaction (*P* > 0.069 in all cases; online supplementary material Table S10).

For the comparison of initial with induced feathers in recaptured birds, we found a significant interaction between treatment and capture time (*F*_2,74_ = 8.443, *P* < 0.001), but no three-way interaction with species (*P* = 0.294), suggesting that the effects were not species dependent. The change in growth bar length from the initial feather to the induced feather was dependent upon feeder availability. There was greater feather growth (9.5% increase in growth bar length) in the induced feather at feeder sites than at control sites, where growth bars were significantly shorter than the original feather (4.2% decrease in growth bar length).

### Disease

Of the pathology observed over the 3 years, 81% of occurrences were avian pox, 8% were conjunctivitis, 6.3% were cloacal infections, and 4.7% were fungal skin disease. From the GLM analysis, we found a significant interaction between treatment and year with regard to the probability of a bird showing symptoms of disease (*F*_1,1387_ = 3.860, *P* = 0.003). We then ran separate GLMs within each of the years and found that birds were more likely to have disease at sites with feeders than at sites without feeders in 2012 (*F*_1,435_ = 3.551, *P* = 0.001) and in 2013 (*F*_1,322_ = 4.304, *P* < 0.001), but not in 2011 (*F*_1,616_ = 0.103, *P* = 0.749; online supplementary material Table S11).

**Figure 4:**
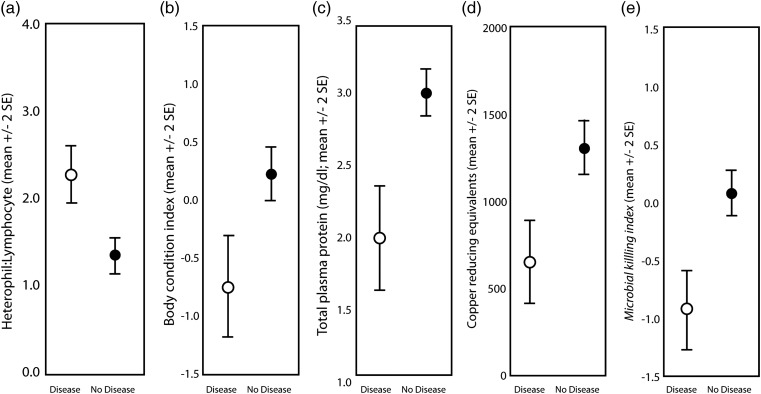
Comparison of heterophil-to-lymphocyte ratio (**a**), body condition index (**b**), total plasma protein (**c**), total antioxidant capacity (**d**) and microbial killing ability index (**e**) for 11 species of feeder-using birds showing symptoms of disease (open circles) and birds without symptoms of disease (filled circles). Differences are statistically significant in all comparisons shown here.

Birds showing symptoms of disease had greater H:L than birds with no symptoms of disease, independent of any other factor (*F*_1,1109_ = 76.575, *P* < 0.001; Fig. 4a). Birds with disease symptoms were in significantly poorer body condition than birds without disease symptoms (*F*_1,1382_ = 15.694, *P* < 0.001; Fig. 4b). Birds with symptoms of disease had lower total plasma protein than birds without disease symptoms (*F*_1,816_ = 4.021, *P* < 0.001; Fig. 4c). There was also a significant main effect of disease on total antioxidant capacity (*F*_1,1221_ = 6.694, *P* < 0.001), with birds showing symptoms of disease having significantly lower antioxidant capacity than those without symptoms (Fig. 4d). Birds showing signs of disease had significantly lower microbial killing ability (*F*_1,1020_ = 5.480, *P* < 0.001; Fig. 4e) than birds with no disease symptoms.

Given the physiological deficits observed in birds showing signs of pathology, we also examined recapture rates of birds captured with disease symptoms compared with recapture rates of birds not showing disease symptoms. Over the entire period of the study, the recapture rate of birds at feeder sites captured with disease was 13.4% and the recapture rate of birds captured with disease at non-feeder sites was 12.1%. Of those recaptured after being captured with disease symptoms at sites with feeders, 50% (12 of 24) no longer showed symptoms of disease, whereas 50% still showed signs of disease. Of those recaptured after being captured with disease symptoms at sites without feeders, 50% (two of four) no longer showed symptoms of disease, whereas 50% still showed signs of disease. No birds in which the symptoms were gone upon recapture were recaptured more than 4 months after the capture in which they showed symptoms of disease. Four months was also the greatest time span between the initial capture and recapture of birds exhibiting symptoms in both cases. No birds that were captured more than once within a given year with symptoms of disease were recaptured in any subsequent years.

## Discussion

Bird feeding significantly influenced the health of birds in Central Illinois, USA over the course of this study. When compared with birds at sites without bird-feeding activity, there were consistent patterns of birds being in greater overall health when feeders were present. However, there were some measures that did not differ between feeder and non-feeder sites, and there were some negative impacts of feeding. Although diseases might be more prevalent at feeder sites, the majority of birds appeared to be better equipped to handle the consequences of disease, because they had lower stress (H:L ratios) and greater antioxidant capacity, demonstrated greater nutritional condition by growing higher-quality feathers, and in general, showed greater innate immune capacity.

Our findings that birds at feeder sites had significantly lower physiological stress (as indicated by H:L ratios) demonstrates that a constant, predictable food source, even if only supplementary to the natural diet, permits investment in costly physiological processes, such as maintenance of a large lymphocyte population. Predictability of food sources has been linked to stress responses in multiple studies (Pravosudov *et al*., 2001; Reneerkens *et al*., 2002; reviewed by Wingfield, 2003), and studies of the effects of food quantity in captive birds have also revealed reduced H:L ratio with increased food availability (see review by Maxwell and Robertson, 1998). This relationship has not held for all studies. Clinchy *et al*. (2004) found that increased food availability did not influence the H:L ratio in free-living song sparrows (*Melospiza melodia*). The H:L ratios were not influenced by supplemental feeding in hooded crows (*Corvus corone*) in Italy (Acquarone *et al*., 2002), nor were they influenced by experimentally variable food availability in captive curve-billed thrashers (*Toxostoma curvirostre*; Fokidis *et al*., 2012). In each of these studies in which a relationship between supplemental feeding and H:L ratio was not found, the experimental manipulation of food was not for such a long duration as that in our study. This physiological response may therefore be influenced by long-term supplementation, but be less sensitive to short-term changes in food availability.

The validity of body condition indices as a true indicator of quality has been questioned on many occasions, and there are many different indices that are used for birds (Labocha and Hayes, 2012). The index we used here was found to be predictive of survival through an epidemic outbreak of Eastern equine encephalitis in Florida scrub jays (*Aphelocoma coerulescens*) in 2008; however, it was not a significant predictor of survival in non-epidemic years (Wilcoxen *et al*., 2010). Given that birds in the present study did show an increase in body condition at feeder sites after a full year of supplemental feeding, we conclude that anthropogenic food had a positive influence on structural size-to-body mass ratio in these birds.

All species of birds need substantial energy to provide fuel for the demanding activities of finding mates, raising young and defending territory. Despite the need for energy, fat reserves may carry costs in terms of winter survival, diminished manoeuvrability and increased predation risk (Lima, 1986). In our study, birds at feeder sites had increased fat deposits even in the spring and summer when migration and winter survival were not challenges the birds were facing (Rogers, 2015). It is possible that the significantly elevated fat deposition in birds at sites with feeders is a negative by-product of the availability of an *ad libitum* food supply that requires very little handling time or effort to consume. Alternatively, the increased fat storage in the birds at feeder sites may have carried relatively little cost and left them better prepared to buffer against environmental perturbations that alter the prevalence of natural food sources. In 2012, our study area experienced extreme drought conditions that persisted for much of the year. It is possible that the increased fat deposits in birds at feeder sites were beneficial amidst such an environmental perturbation that would otherwise reduce availability of natural food sources.

Antioxidant capacity was greater in birds at sites with feeders than in birds without feeders. Though antioxidant levels often vary considerably among bird species, seasons and life-history stages (Cohen *et al*., 2009, and also shown in the present study), antioxidants are critical to the health of all organisms. Fertility, growth, immune function, the development of secondary sexual characteristics and resistance to ageing are all affected by acquisition of antioxidants from food sources (see review by Catoni *et al*., 2008). It seems likely, therefore, that birds at feeder sites, with consistently greater total antioxidant capacity, would be better equipped to handle stressors that lead to the production of free radicals and oxidants, and therefore, be in an overall better state of health than birds without access to supplemental food.

Greater growth bar length in feathers of birds at sites with feeders, in terms of both naturally moulted feathers and feathers induced by experimental removal, indicates a direct nutritional benefit of anthropogenic food to the birds in this study. Other studies with single species or lower numbers of species have also revealed a positive relationship between food availability and feather growth bar length in songbirds (e.g. Grubb, 1989; Waite, 1990; Nilsson *et al*., 1993). One of the more interesting findings from our feather analysis was that birds at sites with feeders showed increased growth bar length when feathers were experimentally removed, but birds at sites without feeders showed decreased growth bar length. This finding alludes to the importance of moulting at the proper time of year. Birds with access to supplemental food may have been able to overcome the cost of regrowing a feather at an improper time by having access to the constant supplemental food source, whereas the birds without feeders were unable to grow high-quality feathers when forced to do so outside of the normal moult schedule.

It is often the case that enhanced immune function can serve either as an indicator of a strong overall health state or as an indicator of an animal facing a consistent pathogen threat. We know that pathogen threat is increased with increased population density (McCallum *et al*., 2001). We observed increased incidence of birds with disease at the sites with feeders when compared with sites without feeders over time. Birds showing clear signs of pathology had significantly reduced innate immune function compared with birds without signs of infection, and birds with greater innate immune defenses showed no deficits in other physiological responses (i.e. no apparent trade-offs). It seems, therefore, that even if the enhanced constitutive immunity in birds at sites with feeders is a product of repeated exposure to pathogens, the birds would have the benefit of a greater first line of immune defense without apparent costs. These costs may have been ameliorated by the constant, predictable food supply provided by feeders. Indeed, multiple studies with captive birds have revealed enhanced innate immune defense with increased food supply (e.g. Klasing, 1998, 2007; Kidd, 2004; Kogut, 2009), which is consistent with the findings in our study of free-living birds.

A long-established ecological principle is that disease transmission occurs at greater rates in populations and communities that are more densely populated than in those that are more sparsely populated (McCallum *et al*., 2001). Bird feeders create a common food source for many birds of the same and different species. Therefore, it is not surprising that we found an increase in disease prevalence at feeder sites compared with non-feeder sites (see also Bradley and Altizer, 2006; Becker *et al*., 2015). In addition, bird feeders can themselves act as fomites, transmitting pathogens from one individual to another (Dhondt *et al*., 2007). Birds with clear signs of pathology showed deficits in most of the physiological metrics in which birds at feeder sites were typically better, but at the peak of infectious disease prevalence, only 8.3% of all birds at feeders exhibited symptoms. Based on the results of PROJECT WILDBIRD^®^, cleaning of bird feeders is not a regular habit among people who feed birds, with 40% of individuals reporting that they cleaned feeders yearly or never at all (Horn and Johansen, 2013). Regular feeder cleaning may reduce disease transmission. In addition to feeder cleaning, it would be interesting to know whether provisioning smaller amounts of food and leaving feeders empty for brief periods (1–2 days) between fillings impacts bird diversity or reduces the overall density of birds at feeders. Any method that reduces density but not diversity, which may therefore reduce disease transmission, would be a worthwhile endeavour for bird-feeding hobbyists who want to reduce negative impacts of feeding while maintaining the species composition of their bird communities at feeders. Future research should aim to explore these options. Conservation organizations should also provide people who feed birds with information on how to clean feeders and its importance in an effort to reduce disease prevalence at feeding stations.

Ten months after the removal of feeders, the health of birds at sites where feeders had been removed did not differ significantly from those sites with no feeder history, suggesting that consistent bird feeding is necessary to maintain the supplemental health benefits of feeding. This result also supports the conclusion that birds are truly using these anthropogenic foods as supplemental sources and are not dependent upon them for their primary sustenance, even after 2 years of unrestricted access to the feeders. Disease prevalence also returned to the pre-feeder frequencies 10 months after feeders were removed, and therefore, increased disease prevalence at feeders does not appear to create persistent, long-term altered disease dynamics in a population. We also found that some of the birds at feeders were capable of clearing or tolerating infections because the symptoms were absent upon subsequent capture. Nevertheless, a small percentage of birds at feeders were clearly suffering from disease, were in extremely poor physiological condition and were not recaptured in a symptom-free state.

One of the limitations of our study is that we do not have estimates of the amount of seed that each individual bird in our study consumed. Recently developed stable isotope analytical techniques have been useful to assess differential use of feeders by birds at sites (Robb *et al*., 2011). Although studies such as that by Robb *et al*. (2011) have shown a great amount of variation in feeder use, even within the same species and among different locations, we are confident that in our study the use of multiple sites with feeders, multiple sample years, a survey of multiple species, collecting data from more than 1000 individuals, and most importantly, the concurrent collection of data at control sites facing the same environmental patterns and perturbations has allowed us to draw strong conclusions regarding the overall impacts of bird feeding on the health of free-living birds.

This study is the first to examine the effects of wild bird feeding on the individual health of a broad range of species across multiple seasons and years. We conclude that birds that use feeders are typically healthier than birds without access to feeders, with the exception of higher disease prevalence rate at feeder sites. In addition, our physiological data suggest that the removal of feeders after they are well established does not lead to a crash in the health state, and as such, feeders appear genuinely to be supplemental and do not create dependency among free-living birds in our area. Studies such as ours can be used to develop evidence-based recommendations that can lead to a better bird-feeding experience for both the people who feed birds and the wild birds themselves.

## Supplementary material

Supplementary material is available at *Conservation Physiology* online.

## Funding

This work was supported by the Wild Bird Feeding Industry Research Foundation; The Scotts Company; Millikin University; Decatur Audubon Society; Illinois State Academy of Science Undergraduate Research Grant; and Sigma Zeta National Math and Science Honor Society Student Research Grants.

## Supplementary Material

Supplementary Data
