# Supplementary material for: Effects of bird-feeding activities on the health of wild birds
Source: Conserv Physiol. 2015 Dec 21;3(1):cov058. doi: 10.1093/conphys/cov058 (PMC4778448; doi:10.1093/conphys/cov058)
Supplement: Supplementary Data [file cov058supp.zip › cov058supp.docx]

**Table 1.** Results from a general linear mixed model with heterophil to lymphocyte ratio as the dependent variable using iterative removal of non-significant variables. The values for each variable below are from the last model in which it was included. Statistically significant variables (p < 0.006) are shown in bold.

| Variable | Num df | Den df | F | p |
| --- | --- | --- | --- | --- |
| **Species** | **10** | **1109** | **16.551** | **0.000** |
| **Treatment** | **1** | **1109** | **18.204** | **0.000** |
| Sex | 3 | 1109 | 0.578 | 0.727 |
| Year | 3 | 1109 | 4.090 | 0.007 |
| **Disease** | **1** | **1109** | **76.575** | **0.000** |
| Age | 4 | 1109 | 0.493 | 0.741 |
| Treatment*Year | 2 | 1107 | 1.849 | 0.151 |
| Year*Disease | 3 | 1104 | 1.904 | 0.127 |
| Species*Treatment | 10 | 1094 | 0.616 | 0.801 |
| Year*Age | 10 | 1084 | 1.082 | 0.373 |
| Species*Sex | 30 | 1024 | 1.162 | 0.088 |
| Species*Year | 30 | 1024 | 1.162 | 0.088 |
| Year*Sex | 7 | 1017 | 1.382 | 0.209 |
| Age*Sex | 12 | 1005 | 1.251 | 0.243 |
| Treatment*Disease | 1 | 1004 | 1.261 | 0.912 |
| Age*Disease | 4 | 1000 | 0.230 | 0.922 |
| Sex*Disease | 3 | 997 | 1.702 | 0.104 |
| Treatment*Sex | 3 | 994 | 0.462 | 0.709 |
| Species*Age | 31 | 963 | 0.398 | 0.999 |
| Treatment*Age | 4 | 959 | 0.324 | 0.862 |
| Species*Disease | 9 | 950 | 0.467 | 0.897 |
| Species*Treatment*Year | 13 | 926 | 0.905 | 0.547 |
| Species*Treatment*Age | 23 | 903 | 1.227 | 0.101 |
| Species*Year*Sex | 43 | 860 | 1.233 | 0.148 |
| Species*Treatment*Sex | 15 | 845 | 1.060 | 0.391 |
| Treatment*Age*Sex | 7 | 838 | 1.315 | 0.240 |
| Species*Age*Sex | 21 | 817 | 0..805 | 0.715 |
| Treatment*Year*Sex | 3 | 814 | 0.241 | 0.868 |
| Year*Age*Sex | 8 | 806 | 0.460 | 0.884 |
| Treatment*Year*Age | 3 | 803 | 0.199 | 0.897 |
| Species*Year*Age | 24 | 779 | 0.341 | 0.999 |
| Species*Age*Disease | 2 | 779 | 0.019 | 0.792 |
| Species*Sex*Disease | 1 | 761 | 0.001 | 0.978 |

**Table 2.** Results from a general linear mixed model with fat score as the dependent variable using iterative removal of non-significant variables. The values for each variable below are from the last model in which it was included. Statistically significant variables (p < 0.006) are shown in bold.

| Variable | Num df | Den df | F | p |
| --- | --- | --- | --- | --- |
| **Species** | **10** | **1279** | **5.650** | **0.000** |
| **Treatment** | **1** | **1279** | **15.151** | **0.000** |
| Sex | 3 | 1279 | 1.020 | 0.383 |
| Year | 3 | 1279 | 1.376 | 0.241 |
| Disease | 1 | 1279 | 0.628 | 0.428 |
| Age | 4 | 1279 | 0.642 | 0.667 |
| Species*Year | 30 | 1249 | 1.650 | 0.060 |
| Species*Age | 33 | 1216 | 1.427 | 0.057 |
| Sex*Age | 10 | 1205 | 1.233 | 0.260 |
| Age*Year | 9 | 1196 | 1.244 | 0.264 |
| Species*Treatment | 10 | 1186 | 0.697 | 0.728 |
| Treatment*Sex | 3 | 1183 | 0.567 | 0.637 |
| Treatment*Year | 2 | 1181 | 0.254 | 0.776 |
| Disease*Age | 4 | 1177 | 0.457 | 0.767 |
| Disease*Species | 9 | 1168 | 0.546 | 0.841 |
| Disease*Year | 3 | 1165 | 0.182 | 0.908 |
| Treatment*Age | 4 | 1161 | 0.175 | 0.951 |
| Species*Sex | 29 | 1132 | 0.599 | 0.955 |
| Disease*Treatment | 1 | 1131 | 0.000 | 0.988 |
| Disease*Sex | 3 | 1128 | 0.098 | 0.961 |
| Sex*Year | 8 | 1120 | 0.268 | 0.976 |
| Species*Treatment*Year | 14 | 1106 | 1.448 | 0.124 |
| Treatment*Sex*Age | 7 | 1099 | 1.399 | 0.202 |
| Species*Treatment*Sex | 25 | 1074 | 1.156 | 0.272 |
| Disease*Species*Sex | 13 | 1061 | 1.194 | 0.278 |
| Disease*Species*Age | 10 | 1051 | 0.917 | 0.517 |
| Disease*Species*Year | 5 | 1046 | 1.149 | 0.332 |
| Species*Age*Year | 42 | 1004 | 1.038 | 0.406 |
| Species*Treatment*Age | 18 | 986 | 0.750 | 0.760 |
| Treatment*Age*Year | 3 | 983 | 0.702 | 0.551 |
| Treatment*Sex*Year | 4 | 979 | 0.375 | 0.826 |
| Species*Sex*Year | 33 | 946 | 0.756 | 0.839 |
| Sex*Age*Year | 8 | 938 | 0.573 | 0.801 |
| Species*Sex*Age | 20 | 918 | 0.528 | 0.956 |

**Table 3.** Results from a general linear mixed model with total antioxidant capacity as the dependent variable using iterative removal of non-significant variables. The values for each variable below are from the last model in which it was included. Statistically significant variables (p < 0.006) are shown in bold.

| Variable | Num df | Den df | F | p |
| --- | --- | --- | --- | --- |
| **Species** | **10** | **1221** | **5.449** | **0.001** |
| **Treatment** | **1** | **1221** | **35.843** | **0.000** |
| Sex | 3 | 1221 | 0.382 | 0.822 |
| Year | 3 | 1221 | 1.400 | 0.241 |
| **Disease** | **1** | **1221** | **6.694** | **0.000** |
| Age | 4 | 1221 | 0.254 | 0.938 |
| Species*Age | 34 | 1187 | 1.368 | 0.079 |
| Disease*Species | 9 | 1178 | 1.878 | 0.051 |
| Disease*Age | 4 | 1174 | 1.492 | 0.098 |
| Disease*Year | 2 | 1172 | 2.009 | 0.135 |
| Species*Year | 29 | 1149 | 1.103 | 0.323 |
| Treatment*Year | 2 | 1141 | 0.933 | 0.394 |
| Treatment*Age | 4 | 1137 | 0.930 | 0.446 |
| Disease*Treatment | 1 | 1136 | 0.437 | 0.509 |
| Species*Sex | 30 | 1106 | 0.895 | 0.631 |
| Species*Treatment | 10 | 1096 | 0.918 | 0.515 |
| Treatment*Sex | 3 | 1093 | 0.620 | 0.602 |
| Sex*Age | 10 | 1082 | 0.490 | 0.910 |
| Age*Year | 10 | 1072 | 0.500 | 0.891 |
| Disease*Sex | 3 | 1069 | 0.159 | 0.924 |
| Sex*Year | 7 | 1061 | 0.425 | 0.906 |
| Disease*Species*Year | 11 | 1050 | 1.862 | 0.057 |
| Species*Treatment*Sex | 24 | 1026 | 1.481 | 0.064 |
| Species*Sex*Year | 42 | 984 | 1.104 | 0.302 |
| Treatment*Sex*Year | 4 | 980 | 1.280 | 0.342 |
| Treatment*Sex*Age | 9 | 971 | 1.069 | 0.383 |
| Species*Treatment*Age | 19 | 952 | 0.954 | 0.515 |
| Treatment*Age*Year | 3 | 949 | 0.114 | 0.952 |
| Species*Age*Year | 33 | 916 | 0.967 | 0.522 |
| Species*Sex*Age | 24 | 892 | 0.925 | 0.568 |
| Species*Treatment*Year | 14 | 878 | 0.705 | 0.771 |
| Sex*Age*Year | 7 | 871 | 0.566 | 0.784 |

**Table 4.** Results from a general linear mixed model with body condition index as the dependent variable using iterative removal of non-significant variables. The values for each variable below are from the last model in which it was included. Statistically significant variables (p < 0.006) are shown in bold.

| Variable | Num df | Den df | F | p |
| --- | --- | --- | --- | --- |
| **Species** | **10** | **1382** | **3.748** | **0.002** |
| **Treatment** | **1** | **1382** | **19.646** | **0.000** |
| Sex | 3 | 1382 | 2.000 | 0.092 |
| Year | 3 | 1382 | 2.349 | 0.052 |
| **Disease** | **1** | **1382** | **15.694** | **0.000** |
| Age | 4 | 1382 | 2.057 | 0.068 |
| **Treatment*Year** | **2** | **1382** | **6.356** | **0.001** |
| Disease*Year | 3 | 1379 | 2.397 | 0.058 |
| Disease*Species | 10 | 1369 | 2.173 | 0.042 |
| Species*Treatment | 10 | 1359 | 1.664 | 0.084 |
| Sex*Year | 8 | 1351 | 1.221 | 0.283 |
| Species*Sex | 30 | 1321 | 1.080 | 0.351 |
| Species*Age | 33 | 1288 | 1.051 | 0.390 |
| Species*Year | 29 | 1259 | 1.293 | 0.138 |
| Disease*Treatment | 1 | 1258 | 1.702 | 0.192 |
| Disease*Age | 4 | 1254 | 1.102 | 0.354 |
| Age*Year | 10 | 1244 | 0.958 | 0.461 |
| Treatment*Age | 4 | 1240 | 0.832 | 0.505 |
| Sex*Age | 10 | 1228 | 0.918 | 0.528 |
| Treatment*Sex | 3 | 1225 | 0.485 | 0.693 |
| Disease*Sex | 3 | 1222 | 0.440 | 0.724 |
| Species*Sex*Age | 41 | 1181 | 0.914 | 0.627 |
| Species*Sex*Year | 43 | 1154 | 1.005 | 0.516 |
| Treatment*Age*Year | 20 | 1138 | 2.640 | 0.062 |
| Species*Year*Age | 43 | 1135 | 0.983 | 0.504 |
| Species*Treatment*Sex | 19 | 1116 | 0.985 | 0.477 |
| Species*Treatment*Age | 17 | 1099 | 1.172 | 0.280 |
| Treatment*Sex*Year | 4 | 1095 | 1.200 | 0.309 |
| Treatment*Sex*Age | 6 | 1089 | 0.999 | 0.425 |
| Species*Sex*Year | 31 | 1058 | 1.043 | 0.404 |
| Species*Treatment*Year | 14 | 1044 | 0.516 | 0.925 |
| Sex*Age*Year | 7 | 1037 | 0.307 | 0.951 |

**Table 5.** Results from a general linear mixed model with microbial killing assay index as the dependent variable using iterative removal of non-significant variables. The values for each variable below are from the last model in which it was included. Statistically significant variables (p < 0.006) are shown in bold.

| Variable | Num df | Den df | F | p |
| --- | --- | --- | --- | --- |
| Species | 10 | 1020 | 0.140 | 0.999 |
| **Treatment** | **1** | **1020** | **19.646** | **0.000** |
| Sex | 3 | 1020 | 1.071 | 0.370 |
| Year | 3 | 1020 | 2.709 | 0.088 |
| **Disease** | **1** | **1020** | **5.694** | **0.000** |
| Age | 4 | 1020 | 0.234 | 0.948 |
| **Treatment*Year** | **2** | **1020** | **5.480** | **0.005** |
| Species*Treatment | 10 | 1010 | 0.339 | 0.971 |
| Species*Year | 29 | 981 | 2.442 | 0.038 |
| Species*Sex | 30 | 977 | 0.776 | 0.801 |
| Sex*Year | 7 | 974 | 2.780 | 0.064 |
| Disease*Sex | 3 | 974 | 0.293 | 0.830 |
| Disease*Treatment | 1 | 973 | 2.809 | 0.094 |
| Age*Disease | 4 | 970 | 0.204 | 0.936 |
| Treatment*Age | 4 | 969 | 0.138 | 0.138 |
| Disease*Year | 3 | 966 | 0.530 | 0.661 |
| Species*Disease | 10 | 960 | 0.212 | 0.995 |
| Treatment*Sex | 3 | 962 | 0.475 | 0.700 |
| Age*Year | 9 | 954 | 0.434 | 0.917 |
| Species*Age | 32 | 928 | 0.440 | 0.997 |
| Sex*Age | 11 | 917 | 0.192 | 0.998 |
| Species*Treatment*Age | 23 | 858 | 1.367 | 0.117 |
| Species*Year*Age | 36 | 822 | 1.220 | 0.178 |
| Species*Treatment*Year | 13 | 809 | 1.235 | 0.095 |
| Species*Treatment*Sex | 21 | 788 | 0.593 | 0.925 |
| Species*Year*Sex | 35 | 753 | 1.014 | 0.139 |
| Species*Age*Sex | 21 | 732 | 1.031 | 0.422 |
| Year*Age*Sex | 9 | 723 | 0.974 | 0.460 |
| Treatment*Year*Age | 2 | 721 | 0.555 | 0.689 |
| Treatment*Year*Sex | 2 | 719 | 0.684 | 0.660 |
| Treatment*Age*Sex | 4 | 715 | 1.305 | 0.252 |

**Table 6.** Results from a general linear mixed model with hematocrit as the dependent variable using iterative removal of non-significant variables. The values for each variable below are from the last model in which it was included. Statistically significant variables (p < 0.006) are shown in bold.

| Variable | Num df | Den df | F | p |
| --- | --- | --- | --- | --- |
| Disease | 1 | 900 | 2.632 | 0.105 |
| **Species** | **10** | 900 | **2.590** | **0.000** |
| Treatment | 1 | 900 | 0.009 | 0.923 |
| Sex | 3 | 900 | 0.830 | 0.506 |
| Age | 4 | 900 | 0.321 | 0.901 |
| Year | 3 | 900 | 1.207 | 0.306 |
| Disease*Species | 9 | 900 | 0.133 | 0.999 |
| Disease*Treatment | 1 | 900 | 0.002 | 0.964 |
| Disease*Sex | 3 | 900 | 1.269 | 0.284 |
| Disease*Age | 4 | 900 | 0.789 | 0.526 |
| Disease*Year | 3 | 900 | 1.166 | 0.322 |
| Species*Treatment | 10 | 900 | 0.915 | 0.519 |
| Species*Sex | 29 | 900 | 1.396 | 0.081 |
| Species*Age | 28 | 900 | 0.708 | 0.869 |
| Species*Year | 29 | 900 | 1.416 | 0.329 |
| Treatment*Sex | 3 | 900 | 0.558 | 0.643 |
| Treatment*Age | 4 | 900 | 0.273 | 0.896 |
| Treatment*Year | 2 | 900 | 1.684 | 0.186 |
| Sex*Year | 7 | 900 | 0.406 | 0.944 |
| Age*Year | 8 | 900 | 0.495 | 0.839 |
| Species*Treatment*Sex | 10 | 900 | 0.061 | 1.000 |
| Species*Treatment*Age | 12 | 900 | 1.007 | 0.435 |
| Species*Treatment*Year | 14 | 900 | 0.732 | 0.721 |
| Species*Sex*Age | 19 | 900 | 0.125 | 1.000 |
| Species*Sex*Year | 19 | 900 | 0.736 | 0.781 |
| Species*Age*Year | 27 | 900 | 1.253 | 0.176 |
| Treatment*Sex*Age | 4 | 900 | .709 | 0.857 |
| Treatment*Sex*Year | 3 | 900 | 0.794 | 0.569 |
| Treatment*Age*Year | 2 | 900 | 1.305 | 0.271 |
| Sex*Age*Year | 6 | 900 | 0.139 | 0.991 |

**Table 7.** Results from a general linear mixed model with total plasma protein as the dependent variable using iterative removal of non-significant variables. The values for each variable below are from the last model in which it was included. Statistically significant variables (p < 0.006) are shown in bold.

| Variable | Num df | Den df | F | p |
| --- | --- | --- | --- | --- |
| Species | 10 | 816 | 0.992 | 0.449 |
| Treatment | 1 | 816 | 0.257 | 0.613 |
| Sex | 3 | 816 | 0.772 | 0.543 |
| Year | 3 | 816 | 0.673 | 0.569 |
| **Disease** | **1** | 816 | **4.021** | **0.000** |
| Age | 4 | 816 | 0.335 | 0.892 |
| Treatment*Year | 2 | 816 | 2.084 | 0.125 |
| Species*Treatment | 10 | 816 | 0.965 | 0.472 |
| Species*Year | 29 | 816 | 1.423 | 0.070 |
| Species*Sex | 30 | 816 | 1.098 | 0.330 |
| Sex*Year | 8 | 816 | 1.327 | 0.226 |
| Disease*Sex | 3 | 816 | 1.251 | 0.290 |
| Disease*Treatment | 1 | 816 | 0.044 | 0.833 |
| Age*Disease | 4 | 816 | 0.867 | 0.483 |
| Treatment*Age | 4 | 816 | 0.704 | 0.589 |
| Disease*Year | 3 | 816 | 0.273 | 0.845 |
| Species*Disease | 9 | 816 | 1.145 | 0.328 |
| Treatment*Sex | 3 | 816 | 0.803 | 0.492 |
| Age*Year | 10 | 816 | 0.913 | 0.616 |
| Species*Age | 30 | 816 | 1.087 | 0.343 |
| Sex*Age | 12 | 816 | 0.980 | 0..466 |
| Species*Treatment*Age | 20 | 796 | 1.305 | 0.111 |
| Species*Sex*Year | 40 | 756 | 1.402 | 0.057 |
| Species*Year*Age | 31 | 725 | 1.575 | 0.078 |
| Species*Treatment*Year | 12 | 713 | 1.096 | 0.360 |
| Treatment*Sex*Year | 4 | 709 | 1.370 | 0.243 |
| Treatment*Sex*Age | 6 | 703 | 1.095 | 0.364 |
| Species*Age*Sex | 20 | 683 | 1.227 | 0.224 |
| Year*Age*Sex | 7 | 676 | 1.168 | 0.319 |
| Species*Treatment*Sex | 9 | 667 | 0.591 | 0.805 |
| Disease*Species*Year | 6 | 661 | 0.278 | 0.947 |
| Treatment*Age*Year | 2 | 659 | 0.001 | 0.999 |

**Table 8.** Results from a general linear mixed model with plasma testosterone level as the dependent variable using iterative removal of non-significant variables. The values for each variable below are from the last model in which it was included. Statistically significant variables (p < 0.006) are shown in bold.

| Variable | Num df | Den df | F | p |
| --- | --- | --- | --- | --- |
| Disease | 1 | 70 | 0.078 | 0.830 |
| Species | 5 | 70 | 0.046 | 0.999 |
| Treatment | 1 | 70 | 0.073 | 0.837 |
| Age | 3 | 70 | 0.042 | 0.976 |
| Year | 2 | 70 | 0.118 | 0.843 |
| Species*Treatment | 7 | 70 | 0.172 | 0.999 |
| Species*Age | 9 | 70 | 0.029 | 0.999 |
| Species*Year | 8 | 70 | 0.034 | 0.999 |
| Treatment*Age | 2 | 70 | 0.022 | 0.874 |
| Treatment*Year | 1 | 70 | 0.135 | 0.969 |
| Age*Year | 3 | 70 | 0.159 | 0.924 |

**Table 9.** Results from a general linear mixed model with plasma estradiol levels as the dependent variable using iterative removal of non-significant variables. The values for each variable below are from the last model in which it was included. Statistically significant variables (p < 0.006) are shown in bold.

| Variable | Num df | Den df | F | p |
| --- | --- | --- | --- | --- |
| Disease | 1 | 80 | 0.907 | 0.344 |
| Species | 5 | 80 | 0.574 | 0.689 |
| Treatment | 1 | 80 | 0.161 | 0.985 |
| Age | 3 | 80 | 0.105 | 0.999 |
| Year | 2 | 80 | 0.032 | 0.981 |
| Species*Treatment | 7 | 80 | 0.146 | 0.999 |
| Species*Age | 9 | 80 | 0.271 | 0.997 |
| Species*Year | 8 | 80 | 0.429 | 0.981 |
| Treatment*Age | 2 | 80 | 0.033 | 0.943 |
| Treatment*Year | 1 | 80 | 0.005 | 0.386 |
| Age*Year | 3 | 80 | 1.029 | 0.362 |

**Table 10.** Results from a general linear mixed model with feather growth bar lengths as the dependent variable using iterative removal of non-significant variables. The values for each variable below are from the last model in which it was included. Statistically significant variables (p < 0.006) are shown in bold.

| Variable | Num df | Den df | F | p |
| --- | --- | --- | --- | --- |
| **Species** | **6** | **998** | **106.423** | **0.000** |
| Treatment | 1 | 998 | 31.12 | 0.000 |
| Sex | 3 | 998 | 0.511 | 0.728 |
| Year | 3 | 998 | 11.522 | 0.000 |
| Disease | 1 | 998 | 0.528 | 0.351 |
| Age | 4 | 998 | 1.113 | 0.349 |
| Treatment*Disease | 1 | 996 | 0.592 | 0.442 |
| Year*Disease | 3 | 993 | 2.251 | 0.081 |
| Species*Disease | 7 | 977 | 1.189 | 0.306 |
| Species*Treatment | 7 | 991 | 1.684 | 0.088 |
| Treatment*Age | 4 | 987 | 0.917 | 0.529 |
| **Treatment*Year** | **2** | **985** | **3.882** | **0.004** |
| Treatment*Sex | 3 | 982 | 0.881 | 0.450 |
| Age*Sex | 11 | 971 | 1.361 | 0.186 |
| Year*Age | 9 | 962 | 1.068 | 0.384 |
| Year*Sex | 8 | 954 | 0.726 | 0.668 |
| Species*Age | 25 | 929 | 0.886 | 0.627 |
| Species*Year | 20 | 909 | 1.027 | 0.391 |
| Species*Sex | 21 | 888 | 0.623 | 0.904 |
| Treatment*Year*Sex | 4 | 883 | 2.184 | 0.069 |
| Species*Treatment*Age | 16 | 868 | 1.227 | 0.090 |
| Treatment*Year*Age | 3 | 865 | 0.993 | 0.395 |
| Year*Age*Sex | 11 | 854 | 0.855 | 0.585 |
| Species*Treatment*Year | 9 | 845 | 0.549 | 0.839 |
| Species*Treatment*Sex | 11 | 825 | 0.723 | 0.717 |
| Species*Year*Age | 29 | 796 | 0.637 | 0.932 |
| Species*Year*Sex | 24 | 772 | 0.555 | 0.959 |
| Species*Age*Sex | 18 | 754 | 0.651 | 0.860 |
| Treatment*Age*Sex | 5 | 749 | 0.396 | 0.852 |
| Treatment*Year*Disease | 2 | 741 | 0.928 | 0.396 |
| Species*Treatment*Disease | 4 | 739 | 1.136 | 0.338 |

**Table 11.** Results from a generalized linear mixed model with disease status (yes or no) as the binomial dependent variable using iterative removal of non-significant variables. The values for each variable below are from the last model in which it was included. Statistically significant variables (p < 0.006) are shown in bold.

| Variable | Num df | Den df | F | p |
| --- | --- | --- | --- | --- |
| Species | 10 | 1387 | 0.820 | 0.610 |
| Treatment | 1 | 1387 | 1.151 | 0.090 |
| Sex | 3 | 1387 | 0.449 | 0.773 |
| Year | 3 | 1387 | 0.019 | 0.889 |
| Age | 4 | 1387 | 2.045 | 0.070 |
| **Year*Treatment** | **1** | **1387** | **3.860** | **0.003** |
| Year*Species | 10 | 1377 | 0.169 | 0.998 |
| Treatment*Sex | 3 | 1374 | 0.265 | 0.851 |
| Year*Species*Treatment | 10 | 1364 | 0.229 | 0.994 |
